# A Novel Prolyl Hydroxylase Inhibitor Protects Against Cell Death After Hypoxia

**DOI:** 10.1007/s11064-013-1175-0

**Published:** 2013-10-17

**Authors:** Satoru Kontani, Eiichiro Nagata, Tsuyoshi Uesugi, Yusuke Moriya, Natsuko Fujii, Toshio Miyata, Shunya Takizawa

**Affiliations:** 1 Department of Neurology, Tokai University School of Medicine, 143 Shimo-Kasuya, Isehara, Kanagawa 259-1193 Japan; 2 Division of Molecular Medicine and Therapy, United Centers for Advanced Research and Translational Medicine (ART), Tohoku University Graduate School of Medicine, Sendai, Japan

**Keywords:** Prolyl hydroxylase inhibitor, Hypoxia, Hypoxia-inducible factor, p53, Prolyl hydroxylase 2

## Abstract

Hypoxia-inducible factor 1 (HIF-1) is regulated by the oxygen-dependent hydroxylation of proline residues by prolyl hydroxylases (PHDs). We recently developed a novel PHD inhibitor, TM6008, that suppresses the activity of PHDs, inducing continuous HIF-1α activation. In this study, we investigated how TM6008 affects cell survival after hypoxic conditions capable of inducing HIF-1α expression and how TM6008 regulates PHDs and genes downstream of HIF-1α. After SHSY-5Y cells had been subjected to hypoxia, TM6008 was added to the cell culture medium under normoxic conditions. Apoptotic cell death was significantly augmented just after the hypoxic conditions, compared with cell death under normoxic conditions. Notably, when TM6008 was added to the media after the cells had been subjected to hypoxia, the expression level of HIF-1α increased and the number of cell deaths decreased, compared with the results for cells cultured in media without TM6008 after hypoxia, during the 7-day incubation period under normoxic conditions. Moreover, the protein expression levels of heme oxygenase 1, erythropoietin, and glucose transporter-3, which were genes downstream of HIF-1α, were elevated in media to which TM6008 had been added, compared with media without TM6008, during the 7-day incubation period under normoxic conditions. However, the protein expression levels of PHD2 and p53 which suppressed cell proliferation were suppressed in the media to which TM6008 had been added. Thus, TM6008, which suppresses the protein expressions of PHD2 and p53, might play an important role in cell survival after hypoxic conditions, with possible applications as a new compound for treatment after ischemic stroke.

## Introduction

Hypoxia-inducible factor 1 (HIF-1) is a post-transcriptionally regulated transcription factor that controls several hypoxia-inducible genes [1–3]. HIF-1 is a heterodimeric transcription factor composed of an oxygen-labile HIF-1α subunit and a constitutively expressed HIF-1β subunit. HIF-1 contains multiple domains, including an oxygen-dependent degradation domain that is responsible for protein stability and transcriptional activity. HIF-1α binding to hypoxic-condition response elements in the enhancers of target genes, such as erythropoietin (Epo) which is one of pleiotropic cytokines [3–6], glucose transporter-1 (Glut-1) [5], vascular endothelial growth factor (VEGF) [5, 7], and p53, increases the expression of molecules regulating the antiapoptotic signaling cascades including Bcl-xL [8], glucose transport, angiogenesis, and cell proliferation, respectively. Thus, HIF-1α is well known to activate genes that protect against tissue damage during hypoxia.

The activity of HIF-1α is modulated by the oxygen-dependent hydroxylation of proline residues by prolyl hydroxylases (PHDs). Specific inhibitors of PHDs might thus be of therapeutic benefit in patients with ischemic heart disease and cerebrovascular attacks.

Some previously reported PHD inhibitors are believed to inhibit the enzyme through an iron-chelating mechanism [9, 10]. Iron-chelating compounds can have a nonspecific binding affinity to iron**-**containing proteins or iron ions and may not be desirable from a therapeutic viewpoint because iron is an essential cofactor for a host of important cellular functions, including oxidative phosphorylation and arachidonic acid signaling.

We recently developed a novel PHD inhibitor, TM6008, that binds to a site that is compensated with l-proline without chelating the iron atom [11]. The aim of the present study was to investigate the effect of TM6008 after hypoxia and to determine how TM6008 affects cell survival after hypoxic conditions capable of inducing the expression of HIF-1α.

## Materials and Methods

### Materials

We used SHSY-5Y cells. The media and reagents used for the cell cultures and transfection were purchased from Invitrogen (CA, USA). A GAS-PAK anaerobic chamber was purchased from BD Biosciences (Cockeysville, MD, USA). We used primary antibodies against HIF-1α (Santa Cruz Biotechnology, Inc., CA, USA), PHD 1 (Novus Biologicals, CO, USA), PHD 2 (Cell Signaling Technology Inc., MA, USA), PHD 3 (Novus Biologicals, CO, USA), VEGF (LAB VISION, CA,USA), Epo (Santa Cruz Biotechnology, Inc., CA, USA), HO-1 (Stressgen, NY, USA), p53 (Cell Signaling Technology Inc., MA, USA), and β-actin (SIGMA-Aldrich, CA, USA).

A TUNEL staining kit (In Situ Cell Death Detection kit) was purchased from Roche Diagnostics, Germany.

### Hypoxic Conditions

We simulated hypoxic conditions using a GAS-PAK anaerobic chamber. After about half the cells in each cell dish had died, the culture dish was removed from the anaerobic chamber and the cells were allowed to recover under normoxic conditions.

In the present study, the control group was incubated under normoxic conditions for 3 or 7 days after being subjected to hypoxic conditions. On the other hand, in the TM6008-added group, 50 μM of TM6008 was added immediately after the cells had been subjected to the hypoxic conditions, and the cells were then incubated under normoxic conditions for 3 or 7 days. We clarified that 50 μM of TM6008 was the most effective concentration for promoting cell survival (Fig. 2c). All the cells were harvested after three or seven days of incubation under normoxic conditions. The cells were then examined using immunocytochemical and western blot analyses.

### Immunocytochemical Analysis

The cells were fixed with 4 % paraformaldehyde (PFA) and were washed using phosphate-buffered saline (PBS). Then, the cells were incubated for 5 min with 0.1 % Triton-X at room temperature. Afterward, the cells were washed 3 times with PBS and were then incubated for 2 h with a blocking solution (4 % normal goat serum in PBS) at room temperature. The cells were allowed to react with the primary antibody (HIF-1α antibody) overnight at 4 °C. The secondary antibody (Rabbit-Alexa594; Invitrogen, CA, USA) was then applied for 2 h at room temperature after the cells had been rinsed 3 times in PBS. Finally, the cells were washed with PBS. The cells were also stained with DAPI (4′,6-diamidino-2-phenylindole) as a nuclear stain. Apoptosis was evaluated using TUNEL staining.

The percentages of HIF-1α-positive and TUNEL-positive cells among 100 cells were determined; all the experiments were repeated at least three times.

### Western Blot Analysis

The cells were homogenized with a homogenizing buffer (50 mM Tris–HCl [pH 7.4], 1 % Triton X_100, 0.5 mM PMSF, 2 mM CaCl_2_, proteinase inhibitor cocktail). Then, the cells were centrifuged at 10,000×*g* for 10 min at 4 °C and the pellets were removed from the samples. The protein concentrations of the samples were determined using a Protein Assay kit (Bio-Rad Laboratories; CA, USA) using bovine serum albumin as a standard. The samples were separated using gel electrophoresis with a 4–12 % gradient. After electrophoretic transfer to a polyvinylidene fluoride (PVDF) membrane (Immobilon-P; Millipore, MA, USA), the membranes were blocked with 4 % bovine serum albumin in PBS. The membranes were then washed and incubated with the primary antibodies at 4 °C overnight. After incubation with the primary antibodies, the membranes were washed with PBS-T (0.1 % Tween 20) and were incubated with the appropriate horseradish peroxidase-conjugated secondary antibodies (Vector Laboratories, CA, USA) for 2 h at room temperature. The membranes were then examined using an enhanced chemiluminescence (ECL) western blotting system (Amersham-Pharmacia, NJ, USA). In the present study, we used primary antibodies against HIF-1α, PHD 1, PHD 2, PHD 3, VEGF, Epo, HO-1, and p53. Equal loading of the proteins was confirmed using β-actin.

### Real-time PCR for Evaluating the Gene Expressions of HIF-1α and PHDs (PHD1, PHD2, and PHD3)

We used real-time PCR to investigate the gene expressions of HIF-1α, PHD1, PHD2, and PHD3. cDNA was transcribed from DNase-treated mRNA (5 mg) using SuperScript III Reverse Transcriptase (Invitrogen, Carlsbad, CA, USA). The TaqMan One-Step real-time PCR Master Mix Reagent Kit (Applied Biosystems, Foster City, CA, USA) was used with each custom-designed, gene-specific primer/probe set to amplify and quantify each transcript of interest. The reactions contained 100 ng of total RNA, 300 nM each of the forward and reverse primers (human HIF-1α [Hs00153153_m1]; human PHD1 (EGLN2) [Hs00363196_m1]; human PHD2 (EGLN1)[Hs00254392_m1], or human PHD3 (EGLN3)[Hs00222966_m1]) for the relevant TaqMan Gene Expression Assays (Applied Biosystems), 200 nM of TaqMan probe, 12.5 mL of 2_ Master Mix without the enzyme uracil DNA glycosylase (UNG), 0.625 mL of multi-scribe and RNAase Inhibitor Mix, and 6.875 mL of RNAase-free water. PCR amplification and real-time detection were performed using an ABI PRISM 7700 Sequence Detection System (Applied Biosystems) for 30 min at 48 °C (reverse transcription), 10 min at 95 °C (AmpliTaq Gold activation), and 38 cycles of denaturation (15 s at 95 °C), followed by annealing/extension (60 s at 60 °C). The data were analyzed using ABI PRISM Sequence Detection Software. β-Actin was used as an endogenous control for the normalization of the input target RNA. Relative quantitation of the real-time PCR data was performed using the comparative threshold (Ct) method [12].

### Statistic Analysis

Each experiment was repeated at least three times, and the results were expressed as the mean ± SD. The statistical analysis was performed using an ANOVA. A value of *P* < 0.05 was considered statistically significant.

## Results

### TM6008 Inhibits Cell Death After Hypoxic Conditions

**Fig. 1:**
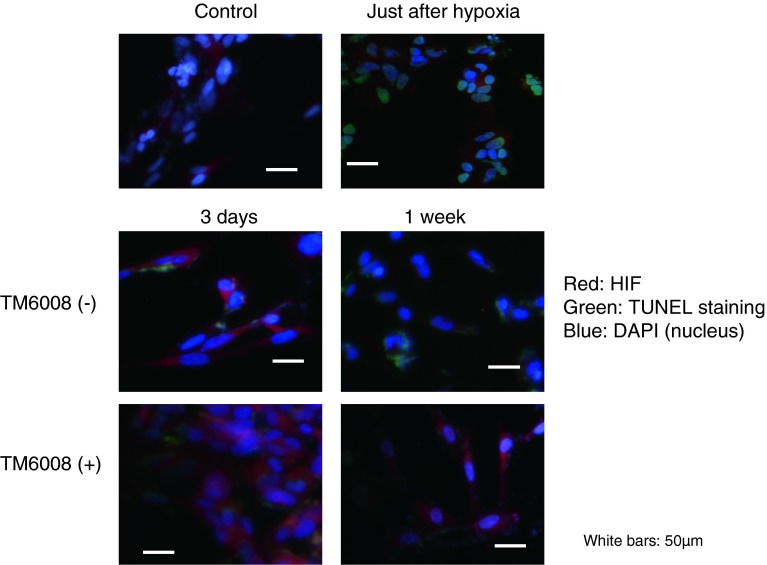
Immunostaining with HIF 1α antibody, TUNEL, and DAPI in SHSY-5Y cells to investigate the effect of TM6008. Just after hypoxia, the numbers of TUNEL**-**positive and HIF**-**positive cells were higher than those in the control in cultures of SHSY-5Y cells. At 3 days after hypoxia, the number of HIF**-**positive cells cultured in the presence of TM6008 was further augmented, compared with the number of HIF-positive cells cultured in the absence of TM6008. Moreover, the number of HIF 1α**-**positive cells was significantly higher after 7 days of culture in the presence of TM6008. Notably, when HIF 1α was activated, it was translocated to the nucleus. Therefore, the* white signal* on the nucleus were shown. However, the number of TUNEL**-**positive cells was lower after 7 days of culture in the presence of TM6008, compared with the number of cells cultured in the absence of TM6008

**Fig. 2:**
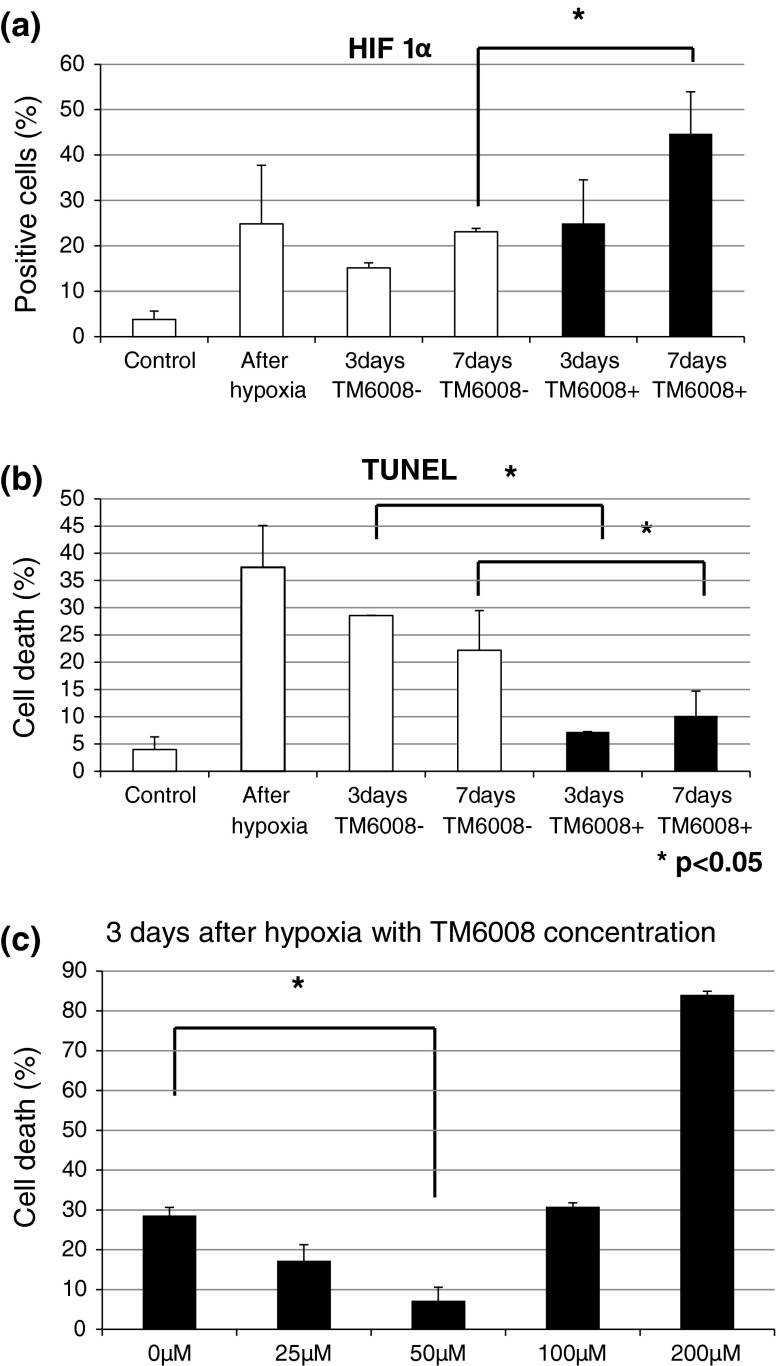
(**a**), (**b**) The numbers of HIF**-**positive cells just after hypoxia and after 3 and 7 days of culture in the presence of TM6008 were larger than that in the control, whereas the number of TUNEL-positive cells just after hypoxia and after 3 and 7 days of culture in the presence of TM6008 were smaller than that in the control. (**c**) Relationship between cell death and the concentration of TM6008. Cell death was significantly reduced in the presence of 50 μM of TM6008, but was increased in the presence of more than 50 μM of TM6008, with a sharp increase observed in the presence of 200 μM of TM6008. The latter dose appeared to be toxic to the cells

The number of HIF-1α-positive cells increased immediately after the hypoxic conditions in both groups (with or without TM6008), compared with the normoxic condition group (*P* < 0.05) (Fig. 1, Fig. 2a, b). The number of HIF-1α-positive cells was also maintained during the 7-day normoxic period after hypoxia (Fig. 2a). Notably, the number of HIF-1α-positive cells was higher for the cells incubated with TM6008 under both conditions (Fig. 2a, *P* < 0.05).

On the other hand, the number of TUNEL-positive cells increased immediately after hypoxia (*P* < 0.05) (Fig. 2b). During the 7-day incubation period under normoxic conditions after hypoxia, the number of TUNEL-positive cells decreased, compared with that just after hypoxia. In particular, the number of TUNEL-positive cells incubated in the presence of TM6008 was significantly lower than that incubated in the absence of TM6008 (*P* < 0.05) (Fig. 2b).

### TM6008 Promotes the Protein Expressions of Genes Downstream of HIF 1α and Suppresses The Protein Expressions of PHD 2, VEGF, and p53

**Fig. 3:**
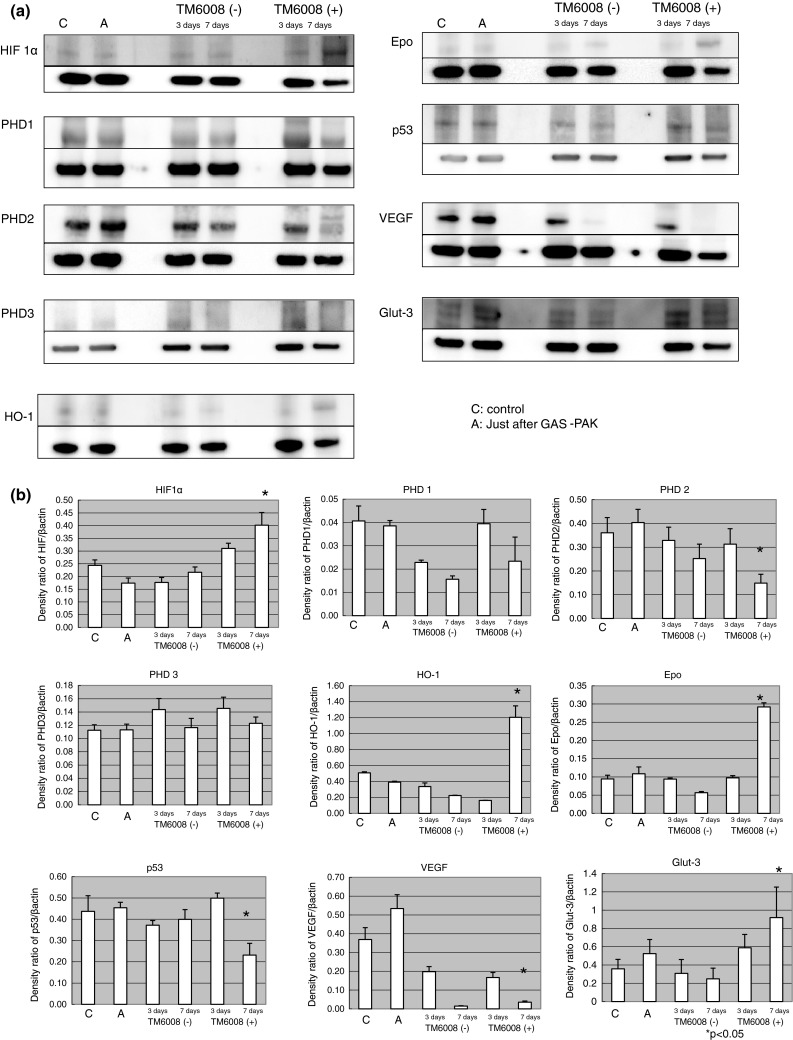

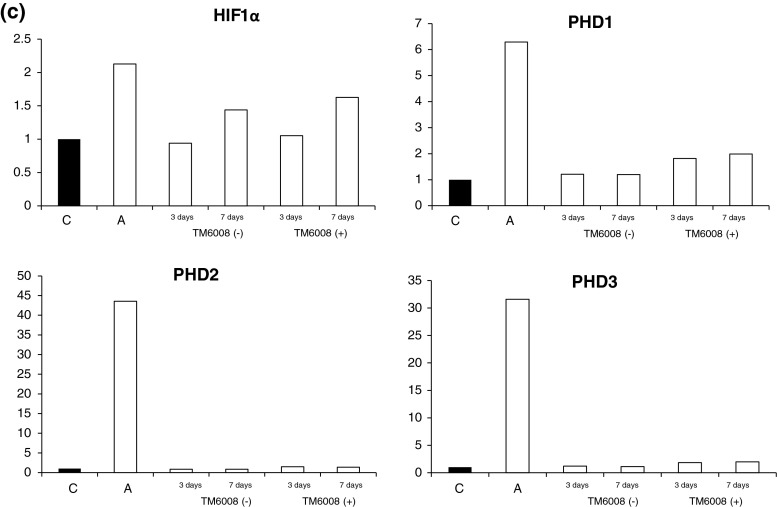
Immunoblottings for HIF and molecules downstream of HIF. (**a**) The protein expression of HIF in cells cultured in the presence of TM6008 at seven days after hypoxia was augmented, compared with that in cells cultured under other conditions. On the other hand, the protein expression of PHD1 in cells cultured in the presence of TM6008 at seven days after hypoxia was decreased, compared with that in cells cultured under other conditions. The protein expressions of PHD2 and PHD3 were increased, similar to the results for HIF. (**b**) The protein expression level of HIF was significantly augmented after 7 days of culture in the presence of TM6008, compared with that in the control. The expressions of HO-1 and Epo were similar to the results for HIF. On the other hand, the protein expression levels of PHD2 and p53 were significantly lower after 7 days of culture in the presence of TM6008. *1* Control, *2* just after hypoxia, *3* without TM6008 after 3 days, *4* without TM6008 after 7 days, *5* with TM6008 after 3 days, *6* with TM6008 after 7 days. *P < 0.05: with TM6008 after 3 days versus with TM6008 after 7 days. (**c**) Quantitative RNA expressions of HIF and PHD genes. Just after hypoxia, all the gene expressions were augmented. However, no significant differences in the RNA expressions were seen between cells incubated with and those incubated without TM6008 during the 3-day and 7-day normoxic periods

Just after hypoxia, the expression levels of genes downstream of HIF-1α, such as HO-1 and Epo, were augmented. Moreover, in the cells that were incubated in the presence of TM6008, the protein expression levels of HIF-1α, HO-1, Epo, and Glut-3 were higher than those under normoxic conditions and after incubation without TM6008 for 7 days. However, the protein expression levels of PHD 2 and p53 in cells incubated in the presence of TM6008 were significantly lower than in those incubated in the absence of TM6008 at 7 days. On the other hand, the protein expression levels of VEGF were reduced at 7 days in cells incubated with or without TM6008 (Fig. 3a, b).

The gene expressions of HIF-1α, PHD 1, PHD 2, and PHD 3 revealed higher expression levels in the samples immediately after the hypoxic conditions in both groups (with or without TM6008), compared with the normoxic condition. Moreover, the expression levels of HIF-1α in the group with TM6008 during the 3-day and 7-day normoxic periods were higher than that in the group without TM6008 during the same periods (Fig. 3c). However, no significant differences in the expression levels of PHDs were seen between the cells incubated with and the cells incubated without TM6008 during the 3-day and 7-day normoxic periods.

These findings suggest that TM6008 might inhibit PHD 2 activity by decreasing the protein expression or degradation of PHD 2.

## Discussion

This study demonstrated that TM6008, a novel specific inhibitor of PHD that we have developed, induces continuous HIF-1α expression under hypoxic conditions and increases the expressions of genes downstream of HIF, such as HO-1, Epo, VEGF, Glut-1, and Glut-3. On the other hand, the effects of TM6008 might also suppress cell death and regulate the protein expression levels of PHDs and p53. In the present study, TM6008 suppressed PHD2, in particular. Three different PHD isoforms have been identified: PHD 1, PHD 2, and PHD 3 [13, 14]. Each PHD has its own tissue and subcellular distribution. PHD 1 is exclusively nuclear, PHD 2 is mainly cytoplasmic but shuttles between the nucleus and cytoplasm, and PHD 3 is present in both the cytoplasm and the nucleus. PHD 2 acts as a decisive oxygen sensor in the HIF-degradation pathway. Although hypoxia decreases the overall PHD activity levels, the upregulation of HIF-1α induces the expression of PHD 2 and PHD 3. This HIF-induced PHD expression ensures the rapid removal of HIF-1α after reoxygenation. Feedback loops may thus exist during hypoxia signaling [15].

The mechanisms of neuroprotection induced by TM6008 may be multifactorial, since HIF regulates a wide range of protective genes such as those involved in erythropoiesis such as angiogenesis (VEGF), Epo, antioxidative stress (HO-1), and glycolysis (Glut-1, Glut-3, and aldolase A). HIF 1α is also known to activate the expression of VEGF. In this study, since we used SHYSY-5Y cells, whether an elevation in VEGF expression might have occurred was unclear because these cells might not contain any vasculogenesis factors.

Epo is produced and released mainly by the kidneys and acts on the bone marrow to promote the proliferation of erythrocytes [16]. During hypoxic conditions, Epo is upregulated as one of the many gene products whose transcription is stimulated by HIF 1α [17]. Epo is not the only gene product whose expression is increased by HIF 1α. Many other proteins are upregulated during hypoxia and are also involved in iron/heme metabolism, including HO-1. During hypoxia, coordination of the upregulation of Epo and the induction of HO-1 is critical for the development of polycythemia.

Our previous study revealed the upregulation of Glut-3 by TM6008 in a gerbil forebrain ischemia model (data not shown). The present results also showed the upregulation of Glut-3 in response to treatment with TM6008 [11]. Although Glut-3 is known to protect against a decline in brain glucose uptake under ischemic conditions [18, 19], whether or not Glut-3 contributes to neuroprotection in cell cultures under hypoxic conditions is unclear. Of interest, we noted the expression of p53 in the cell cultures in the present study. An increase in p53 expression after cell injury stops the cell cycle and leads to apoptosis [20]. p53 is known to control abnormal cell proliferation and to suppress cancer proliferation. Thus, the present study also suggested that TM6008 may protect against cell death and may accelerate cell survival in neurons after an ischemic stroke.

However, HIF-1α might also promote cell proliferation; in other words, it might induce tumorigenesis. We have not yet examined the optimal timing for the administration of TM6008 so as to enable cell survival without causing tumorigenesis, but tumorigenesis may be unlikely to occur if TM6008 is administered within a week after the onset of ischemic stroke in humans.

In the present study, we administered TM6008 to cell cultures after subjecting them to hypoxic conditions and observed a cell protective effect. These results suggest that it might be feasible to rescue cells after hypoxic cell damage. Practically speaking, the rescue of patients who have suffered an ischemic stroke may be possible using TM6008 in the future.

In conclusion, TM6008 might inhibit translation of PHD 2 gene or degrade PHD 2 protein.
